# Social context and daily fluctuations in adolescents’ mindset: the role of parental beliefs, appraisal of increasing marks, and feedback

**DOI:** 10.1007/s10212-025-01009-6

**Published:** 2025-10-06

**Authors:** Ilona M. B. Benneker, Fanny de Swart, Nikki C. Lee, Nienke M. van Atteveldt

**Affiliations:** 1 Section of Clinical Developmental Psychology & LEARN! Research Institute, Vrije Universiteit, Amsterdam, The Netherlands; 2 Department of Developmental Psychology, Utrecht University, Utrecht, The Netherlands; 3 Mencia de Mendozalyceum, Breda, The Netherlands

**Keywords:** Diary study, Parent–adolescent interaction, Feedback, Mindset

## Abstract

Mindset is generally conceptualized as a stable trait, but recent research suggests that the social context may play a pivotal role in its development and adjustment (de Ruiter & Thomaes, 2023; King, 2020); Lou & Li, 2023). Empirical investigations have primarily focused on the social context of teachers and peers with less attention to the role of parents. This study seeks to explore the relationship between parents and their adolescents’ intelligence mindset, by examining the effects of parents’ intelligence mindset, failure beliefs, and appraisal of increasing academic marks, as well as the daily feedback they provide, using a combination of cross-sectional and daily diary methods. The results of the cross-sectional study, from a sample of Dutch adolescents (*M*_age_ = 14.47 years) and their parents (*M*_age_ = 47.60 years) revealed that a more growth-oriented intelligence mindset in parents relate to a more growth-oriented intelligence mindset in their adolescents. Furthermore, parents’ result-oriented day-to-day feedback was found to be negatively associated with adolescents’ intelligence mindset, demonstrating that a focus on school marks may inhibit the development of a growth mindset in adolescents. These findings have useful implications, such as providing new insights into the dynamic interplay between parents’ intelligence mindset, the day-to-day feedback they provide, and their adolescents’ intelligence mindset, which may be important factors for adolescents’ learning attitudes and academic success.

## Introduction

The declining academic motivation in adolescents is concerning, as motivation is vital for school success (see e.g., Gnambs & Hanfstingl, 2016; Gottfried et al., 2001; Scherrer & Preckel, 2019). An important determinant of adolescents’ motivation and achievement is the implicit belief that they hold about the malleability of their intelligence, also called mindset (Burnette et al., 2013, 2023; Dweck & Molden, 2005; Sarrasin et al., 2018). Adolescents who believe that their abilities are malleable, i.e., have a growth mindset, typically demonstrate higher achievement and more motivation for learning (Benneker et al., 2023; Burnette et al., 2018; Yeager et al., 2019).

To date, mindset has mainly been approached as an individual and rather stable characteristic (see e.g., Burnette et al., 2013; Dweck & Molden, 2005; Sisk et al., 2018). Recently, both theoretical models and empirical research on mindset increasingly acknowledges that the social context (i.e., parents, teachers, and peers) might be important in shaping mindset (de Ruiter & Thomaes, 2023; King, 2020; Lou & Li, 2023; Yu et al., 2022), and that mindset might also have a situational component that fluctuates over time (de Ruiter & Thomaes, 2023). However, until now, empirical studies have focused mainly on the social context of teachers and peers and much less on the role of parents. Additionally, there seem to be no studies that empirically assess fluctuations in mindset across days. Therefore, the current study will first investigate the role of parental beliefs (about intelligence and failures) and the way they appraise adolescents’ achievements in shaping adolescents’ mindset; and second, how parental feedback is related to short-term daily fluctuations in adolescents’ mindset.

### Parental beliefs and adolescents’ mindset

Decades of research emphasized mindset as an important individual characteristic, but recently, the role of the social context has increasingly gained attention. Mindsets related to intelligence can range from believing that abilities are unchangeable (fixed) and cannot be improved, to believing that they are malleable (growth) and can be improved (Burnette et al., 2013; Dweck & Molden, 2000). There is much empirical research showing that adolescents with a growth mindset often have higher intrinsic motivation (Burnette et al., 2013; Dweck & Molden, 2005; Janssen et al., 2022; Liu, 2021; Renaud-Dubé et al., 2015), which remains stable under challenging circumstances (Benneker et al., 2023) and relates to adolescents’ school achievements. Adolescents with a more fixed mindset, however, frequently show lower intrinsic motivation and academic performance. Only recently, there has been more attention given to the social context in which mindsets may develop (de Ruiter & Thomaes, 2023; King, 2020; Lou & Li, 2023; Yu et al., 2022). In their process model of mindsets (PMM), de Ruiter and Thomaes (2023) formulated a theoretical model in which mindset beliefs, behaviors, and situational responses and appraisals are considered within interactions with significant others in order to better understand the underlying dynamics of how mindsets develop. This theory is novel because, for one, it assumes a larger role of the social context such as teachers, parents, and peers in the development of mindset. Secondly, according to this theory, mindset also has a situational component that fluctuates over time. As a result, mindsets should not be seen as fixed traits that exist within individuals, but as a more dynamic property shaped by ongoing interactions between individuals within a certain (social) context.

Even though the PMM theory should be broadly applicable to all social contexts, empirical evidence is derived mainly from research on teachers. As such, it is important to gain more insight into its applicability to parental influences as a social context. During adolescence, the role that parents play when raising their adolescents becomes more focused on supporting and guiding (O’Connor et al., 2012; Van de Werff, 2017). It is possible that, due to their parental role and their authority, parents may have a strong influence on their adolescents’ beliefs and behaviors (Liu et al., 2023; Nogueiras et al., 2017). At the same time, adolescents have a tendency toward “social tuning,” which means that they adapt their beliefs to be the same as those with whom they want to strengthen a social bond (Sinclair et al., 2005). Parents, therefore, are able to create an environment that their adolescents want to adapt to (de Ruiter & Thomaes, 2023). This might also be explained by the expectancy effect (e.g., Frome & Eccles, 1998; Jodl et al., 2001), which shows that parents’ perceptions of their children’s abilities predict their children’s own evaluation of their abilities.

During adolescence, adolescents need to develop their own values and beliefs (Dent & Koenka, 2016; Wesarg-Menzel et al., 2023) and parental beliefs, such as their mindset and their failure beliefs, are an important source of influence for their adolescents through these changes. For example, parents who hold a more growth mindset are more likely to have adolescents who adopt mastery-oriented goals, whereas those with a fixed mindset tend to raise children who pursue ability-focused and performance goals (Kim et al., 2024). In addition to influencing goal orientation, parental mindset has been positively associated with adolescents’ persistence (Song et al., 2022). Furthermore, an investigation by Sheffler & Cheung (2024) demonstrated that mothers with growth mindsets engaged in more supportive parenting behaviors, such as providing constructive guidance during learning tasks. These behaviors, in turn, fostered adolescents’ development of growth-oriented learning practices. Altogether, this provides substantiation that parents’ mindset may be important for adolescents’ mindset development.

This means that parents with a fixed mindset may raise their adolescents with the understanding that intelligence is unchangeable, which may be concerning as it consequently may lead to their adolescents developing a fixed mindset. Empirical studies that focused on the direct relation between parents’ mindsets and their adolescents’ mindsets are scarce, and they mainly target younger groups and show varying results. A previous study by Tian (2019) suggested that parents’ own mindset can be transmitted to their children. This is in line with other investigations in which parents’ growth mindset may be associated with growth mindset-related characteristics in their adolescents, such as high persistence (Jose & Bellamy, 2012), high motivation (Pomerantz & Dong, 2006), and high expectations of their own abilities (Rattan et al., 2012). Contrarily, other studies found no evidence for the relationship between parents’ mindset and their children’s mindset (Gunderson et al., 2018; Haimovitz & Dweck, 2016). These varying results show that there is no general agreement yet on the relationship between parents’ and their adolescents’ mindset (Gunderson et al., 2018; Haimovitz & Dweck, 2016; Tian, 2019).

Not only the mindset of parents may relate to adolescents’ mindset, but how parents view failure to achieve might be important as well. Previous investigations showed that the mindset of children may be predicted by their parents’ attitudes toward failure. Parents who viewed failure as debilitating more often had children who developed a fixed mindset, while parents who viewed failure as a learning opportunity more often had children with a growth mindset (Haimovitz & Dweck, 2016; Matthes & Stoeger, 2018; Tao et al., 2022). These investigations suggest that parents’ failure beliefs may be very important for the development of their children’s mindset.

### The mediating role of appraisal of increasing marks

Parental beliefs may not only directly relate to the development of their adolescents’ mindset, but could also indirectly affect mindset through the subtle messages they communicate to their children (Jodl et al., 2001). An example of such a subtle message could be how parents appraise the achievements of their adolescents. Literature identifies two distinct forms of appraisals, namely, result-focused appraisals in which individuals emphasize actual school achievements, and process-focused appraisals in which practice and the learning process are valued more than school achievements. A study by de Kraker-Pauw et al. (2017) in teachers showed that teachers with a more growth mindset valued increasing marks, regardless of whether these marks were sufficient or insufficient, more than teachers with a stronger fixed mindset. Growth mindset teachers, therefore, seem to focus more on the process of improvement and show more positive appraisal of increasing marks. Other studies with teachers demonstrated that teachers with a focus on increasing school achievements enhance adolescents’ motivation (Meece et al., 2006; Wilbert & Grunke, 2010). Given the influence of parents on their developing adolescents, it is possible that such appraisals by parents are also important influences. Parents with a more growth mindset or those who believe that failure is enhancing may appraise their adolescents’ increasing marks more than the absolute results, which may in turn encourage their adolescents to develop a growth mindset and consequently increase their motivation.

### Parental feedback and daily fluctuations in adolescents’ mindset

According to the recently developed PMM theory of de Ruiter and Thomaes (2023), mindset is not a fixed trait within an individual, but may fluctuate depending on the ongoing interactions between an individual and their environment. Even though day-to-day fluctuations in mindset have, to our knowledge, never been investigated, these fluctuations have already been established in various forms of motivational beliefs and achievement goals (Klootwijk et al., 2021; Kramer et al., 2024; Neubauer et al., 2022; Waninge et al., 2014). Adolescents try to understand their experiences while interacting with peers, teachers, or their parents. When they are presented with views that are different from their own, this will provide them with a chance to adapt their own views, which could be key to the process of possible fluctuations in mindsets.

Importantly, when these interactions take place between an adolescent and their parent, the adult might have a stronger impact on the adolescent than a peer (de Ruiter & Thomaes, 2023) due to their role and authority, and to adolescents’ tendency to match their beliefs with someone they want to maintain a bond with (Sinclair et al., 2005). This underlines the importance of carefully considering how daily communication between parents and their adolescents takes place. An important factor in this communication could be the feedback on school experiences that parents provide to their adolescents. According to the PMM theory of de Ruiter and Thomaes (2023), feedback may be essential for the development and adjustment of mindsets. Related to the above-mentioned appraisal of increasing marks, studies have also shown that providing children with positive feedback for perseverance and hard work, also called process-focused feedback, may focus on remedies for their lack of success and search for new strategies to find a solution for their problem. Therefore, they may come to believe that intelligence is malleable (growth mindset). Children who receive feedback solely on their school achievements, also called result-focused feedback, may focus on the cause of their failure and conclude that they are not smart enough and therefore come to believe that intelligence is unchangeable (fixed mindset) (Gunderson et al., 2013; Mueller & Dweck, 1998; Zentall & Morris, 2010).

Through the occurrence of these amplification processes, mindsets may develop and change over time in interaction with their parents’ feedback (de Ruiter & Thomaes, 2023). Surprisingly, to the best of our knowledge, there are no investigations that zoom in on the daily fluctuations in parental feedback and their relationship with short-term changes in adolescents’ mindset. There are some studies on parental feedback across long time intervals (years) that showed that parental feedback is important for children’s development. For example, a longitudinal study with two waves over 1–2 years showed that toddlers who received process feedback developed a growth mindset (Gunderson et al., 2018), while another study showed that parents who provide their children (14–38 months) with positive feedback on their effort, was associated with their children’s persistence at age 7–8 year old (Gunderson et al., 2013). Moreover, there is some scarce evidence that parental feedback fluctuates from day to day and that this might have consequences for their children. For example, on stressful days, parents reported being less encouraging of their children’s engagement in physical activity (Dunton et al., 2019) with negative results for their children’s behaviors. Another study found that daily parental feedback was associated with increased persistence in children on a specific task (Leonard et al., 2022). To get a better understanding of this daily parental impact on adolescents’ development, it is important to investigate the day-to-day parental feedback and its relationship with fluctuations in adolescents’ mindset.

### Current study

In this study, we aimed to investigate the role of parental intelligence mindset and failure beliefs, their appraisal of increasing marks, and the feedback they provide on a daily basis, on their adolescents’ mindsets in a combined cross-sectional and daily diary study with parent–adolescent dyads. Specifically, we address two key research questions in the cross-sectional study: (1) “How do parents’ mindset and failure beliefs relate to their adolescents’ mindset?” and (2) “Is this relationship mediated by parents’ appraisal of increasing marks?” Based on previous studies with conflicting and sometimes weak evidence, we cautiously hypothesize a positive relationship between parents’ growth mindset and their adolescents’ growth mindset (Tian, 2019). We expect a negative relationship between parents’ failure beliefs and their adolescents’ mindset (Haimovitz & Dweck, 2016; Matthes & Stoeger, 2018). Furthermore, we hypothesize that parents’ appraisal of increasing marks mediates the relationship between parents’ mindset and their adolescents’ mindset (Kraker-Pauw et al., 2017; Meece et al., 2006; Wilbert & Grunke, 2010). Parents with a more growth mindset or who see failure as an opportunity to learn, may value increasing marks, and therefore value the process leading to (academic) achievements more than the actual result. They may display this via subtle messages in their communication, and it may, in turn, stimulate their adolescents to develop a growth mindset (Kraker-Pauw et al., 2017). In the diary study, we address one key research question: (3) “How does received process- or result-focused feedback during day-to-day interactions with parents relate to adolescents’ daily mindset?” We hypothesized that process-focused feedback provided by parents during day-to-day interactions may relate to a more growth mindset in adolescents on those days, while result-focused feedback during day-to-day interactions may relate to a more fixed mindset in adolescents on those days (Gunderson et al., 2018; Haimovitz & Dweck, 2016).

## Methods

This study’s variables, hypotheses, and planned analyses were preregistered on Open Science Framework (https://osf.io/y6eag/?view_only=8cfecbc49db845ba92f869a45f110430).

### Participants

Participants in this study were Dutch adolescents and one of their parents, recruited from two different secondary schools in the southern part of the Netherlands. The researchers contacted schools interested in participating in scientific research to ask for permission to send recruitment information to adolescents and their parents. The two schools that agreed to participate were asked to forward an email with information about the research project to all parents.

For the first two research questions, we collected the data of a total of 131 adolescents and 133 parents. We were unable to match the data of two adolescents with their parents, and we were also unable to match the data of four parents with their adolescents. We therefore excluded the two adolescents and four parents, leaving us with 129 adolescent–parent dyads. The mean age of the adolescents (53% female) was 14.47 years (SD = 2.01), and the mean age of the parents (73% female) was 47.60 years (SD = 4.58) (see Table 1 for descriptive statistics).

**Table 1:** Descriptive statistics of adolescents and parents: age, gender, and level of education

	Adolescents (*n* = 129)	Parents (*n* = 129)
Gender (f:m)	68:50 (unknown:11)	94:30 (unknown:5)
Age		
Mean (SD)	14.47 (2.01)	47.60 (4.58)
Range	11–19 years	34–62 years
Level of education^a^		Highest level of education (%)
Primary school		1 (0.78)
Secondary school		5 (3.88)
MBO		7 (5.43)
HBO		60 (46.51)
WO		49 (37.98)
Other		2 (1.55)
Missing		5 (3.88)^a^

^a^The Dutch schooling system after secondary school is divided into MBO (secondary vocational education), which is focused on vocational training, HBO which focused on higher professional education/higher vocational education and WO (academic education, i.e., university)

For the third research question, we included parent–adolescent dyads who completed 60% or more of the daily measurements (Griffiths et al., 2022). One adolescent and six parents missed more than 40% of the daily measurements, and these parent–adolescent dyads were excluded from further analysis. A total of 122 adolescent–parent dyads were therefore included in the diary analysis. Adolescents received a monetary reward of 5 € per week if they missed a maximum of two (of seven) measurements per week. In total, they could receive 10 €. Parents did not receive a monetary reward.

### Procedure

Prior to the study, adolescents and their parents gave written informed consent, and all procedures were approved by the Vrije Universiteit Amsterdam, Faculty of Behavioral and Human Movement Sciences ethics committee. Adolescents and their parents used their mobile devices to fill in the questionnaires. In February 2020, adolescents completed a questionnaire on their intelligence mindset, while parents finished a questionnaire on their intelligence mindset, failure beliefs, and appraisal of increasing marks. Demographic measures, including gender and age, were also included in these questionnaires. Two weeks later, the daily diary study started. Adolescents completed a questionnaire on their intelligence mindset on a daily basis for 2 weeks, while parents completed daily questionnaires on the type of feedback they provided to their adolescents. Our diary study was carried out for 14 days during both school days and weekend days. Adolescents and their parents received a message at 1800 h in the evening. They were given 2 h to fill in the diary questionnaire. At 1930 h (after 1.5 h), they received a reminder to fill in the diary questionnaire.

Our study, however, focuses on parental feedback in relation to school achievements; therefore, school days were better suited to answer the research question than weekend days. Additionally, school days may be more similar to each other and therefore more comparable. Based on these arguments, we decided to exclude the weekend days from our analysis. We included a total of ten school days for each participant in our final analysis.

## Measurements

### Cross-sectional measurements

#### Adolescents’ intelligence mindset (measured in adolescents)

Intelligence mindset in adolescents was measured in the cross-sectional study (first research question) using the Implicit Theories of Intelligence Scale of Dweck (1999), which was adapted by De Castella and Byrne (2015). The scale consists of eight items (four fixed mindset items and four growth mindset items). The answers were scored on a Likert scale from 1 to 6. A score of 1 meant “strongly disagree,” while a score of 6 meant “strongly agree.” The fixed mindset items were reverse-scored, and the mean of the eight items was calculated. A higher score meant having a stronger growth mindset, while a lower score meant having a stronger fixed mindset. An example of a growth mindset statement from this cross-sectional study is “Regardless of my current intelligence level, I think I have the capacity to change it quite a bit” and an example of a fixed mindset statement from this cross-sectional study is “To be honest, I don’t think I can really change how intelligent I am.” The scale showed excellent internal consistency (McDonald’s *ω* =.92).

#### Parents’ intelligence mindset (measured in parents)

Parents’ intelligence mindset was measured using the same questionnaire as that for adolescents’ intelligence mindset for the cross-sectional study (first research question). Here, too, the scale that showed internal consistency of the scale was excellent (McDonald’s *ω* =.93).

#### Parents’ failure beliefs (measured in parents)

Parents’ failure beliefs were assessed using the parents’ failure mindset scale (Haimovitz & Dweck, 2016). The scale consists of six items (three failure is debilitating items, and three failure is enhancing items). The answers were scored on a Likert scale from 1 to 6. A score of 1 meant “strongly disagree,” while a score of 6 meant “strongly agree.” The three failure is debilitating items were reverse-scored, and the mean of the six items was calculated. A higher score meant that failure was seen as enhancing, while a lower score indicated a more debilitating view of failure. An example of a failure is enhancing item is “Experiencing failure enhances my performance and productivity” and an example of a failure is debilitating item is “Experiencing failure inhibits my learning and growth.” The scale showed excellent internal consistency (McDonald’s ω =.93).

#### Appraisal of increasing marks (measured in parents)

We built on the adaptations of Kraker-Pauw et al. (2017) to the original Reference Norm orientation test (Rheinberg, 1980) to assess appraisal of achievement of parents. We made several additional adaptations to the test to make it better suited for our goal of investigating the appraisal of increasing marks. These adaptations included the elimination of the three items that were stable and the addition of two sets with increasing marks and one set with decreasing marks. In total, the test consisted of 12 sets of 3 subsequent marks: 6 sets with increasing marks and 6 sets with decreasing marks. We kept the interval the same between the different marks of one set (see Table 2). Every third mark of a set occurred in two sets, once in a decreasing set and once in an increasing set. Parents had to evaluate the third mark of a total of 12 sets of sequential marks obtained by fictional adolescents. They had to rate this last mark of a student on a scale from – – (poor achievement) to + + (good achievement) with +/– meaning a neutral evaluation. The scale was rescored into the following values: – – (1), – (2), +/– (3), + (4), and + + (5); higher score representing a higher appreciation of the final mark. Parents who evaluated the third mark of an increasing set as very good are suggested to value process over result; when they evaluated this less positively, they could be more result-oriented (Kraker-Pauw et al., 2017). To get a score on the appraisal of increasing marks, we took the average of the six sets with increasing marks. The sets with decreasing items were included for heterogeneity of the sets. The scale showed good internal consistency (McDonald’s *ω* =.89).

**Table 2:** Three sequential marks for 12 fictional students. The third mark had to be evaluated

Student	Mark 1	Mark 2	Mark 3		Participant’s appraisal of mark 3 (– –, –, +/–, +, + +)
1	4.0	4.5	5.0	→	
2	1.5	2.0	2.5	→	
3	5.0	4.5	4.0	→	
4	6.5	7.0	7.5	→	
5	3.0	3.5	4.0	→	
6	5.0	5.5	6.0	→	
7	6.0	7.0	8.0	→	
8	4.5	3.5	2.5	→	
9	7.0	6.0	5.0	→	
10	9.0	8.5	8.0	→	
11	7.0	6.5	6.0	→	
12	9.5	8.5	7.5	→	

### Daily diary study

Since participants had to fill out the same questionnaire on a daily basis, the daily measures were kept brief in order to promote compliance (Palmier-Claus et al., 2011).

#### Adolescents’ intelligence mindset (measured in adolescents)

In the diary study (second research question), intelligence mindset was measured for 10 days using the original mindset questionnaire (Carol et al., 1995), consisting of only three fixed mindset items. We adapted the questions to make them suitable for that specific day. As in the cross-sectional study, answers were scored on a Likert scale from 1 to 6. In order to make the score on daily mindset comparable to the score on mindset in the cross-sectional study, all the items were reverse-scored, and the average was calculated. A higher score meant having a stronger growth mindset on that day. An example of a fixed mindset statement is the diary study is “Today, to be honest, I don’t think I can really change how intelligent I am.” The scale showed good to excellent internal consistencies (McDonald’s *ω* =.88 to.95).

#### Parental feedback (measured in parents)

We measured self-reported parental feedback for 10 days with eight items about how often they had given certain types of feedback to their adolescent that day.

Four items focused on process-oriented feedback, and four items focused on result-oriented feedback. Of the four process-oriented and four result-oriented items, two were positively stated (e.g., “You used good (learning) strategies for that task”) and two were negatively stated (e.g., “You did not use good (learning) strategies for that task”). In the instruction, we explicitly mentioned that parents did not literally have to say the sentence. The answers of the parents were scored on a Likert-scale from 1 to 4, in which 1 meant that the parent had not said this during the day on most days, 2 meant that the parent had said this 1 or 2 times during that day, 3 meant that the parent had said this 3–4 times per day, and 4 meant that the parent had said this 5 times or more per day. We rescored these answers into the following scale: (1) this was not said during the days, (2) this was said sometimes during the days, (3) this was said regularly during the days, and (4) this was often said during the days. A preliminary scan of the collected data showed that the negatively stated items were mostly scored with a 1. This meant the parent had not said these items during the days. Based on this and on the fact that we were more interested in how process feedback could be related to a growth mindset, we decided to focus solely on the positively stated feedback items. For the process-oriented feedback, the mean score of the two positively stated process-oriented feedback items was calculated. For the result-oriented feedback score, the mean of the two positively stated result-oriented feedback items was calculated. The two-item scale on result-oriented feedback showed strong internal consistency, as indicated by daily inter-item correlations between *r* =.36 to *r* =.63. The two-item scale on process-oriented feedback also showed strong internal consistency, as indicated by daily inter-item correlations between *r* =.48 to *r* =.64.

## Analysis

Statistical analyses of the first research question were performed using the psych package (Revelle & Revelle, 2015) and the ROBMED package (Alfons et al., 2022a, 2022b) of the open-source program R (version 4.0.2) (R Core Team, 2020). For the second research question, we used the lme4 package (Bates et al., 2015; Bryk & Raudenbush, 1992) in R for hierarchical linear modeling. Descriptive statistics were used to assess demographic data such as gender, age, and level of education. Correlations among all study variables were calculated using Pearson’s correlation for continuous variables in the cross-sectional study.

For the cross-sectional design (research question 1), we first investigated the direct relationship between parental intelligence mindset and adolescents’ mindset and between parental failure beliefs and adolescents’ mindset. We checked for the presence of multicollinearity among the predictor variables using the variance inflation factor (VIF). According to established guidelines, a VIF value greater than 5 indicated problematic multicollinearity (Neter et al., 1983). In our analysis, all VIF values ranged from 1.007 to 1.191, suggesting that multicollinearity was not a concern. Other assumptions were checked and found to be satisfactory as well.

Gender and age of the adolescent and gender of the parent were included as covariates. Next, we conducted a mediation analysis, with appraisal of increasing marks as the mediator between parental intelligence mindset and parental failure beliefs and adolescents’ mindset. The path coefficients were in standardized form (*β*). To assure the validity of the mediation effects in an analysis with a relatively small sample size, the ROBMED package (Alfons et al., 2022a, 2022b) was applied. It uses the robust MM-estimator and fast-and-robust bootstrap methodology (5000 samples), which accounts for non-normality in the data and produces robust estimates (Alfons et al., 2022a).

In the diary study (research question 2), preliminary inspection of the data showed that a total of 8.28% of the data points of adolescents and 7.42% of the data points of parents were missing. Since missingness was lower than 10% (Bennett, 2001), we accounted for missing data by utilizing multiple imputation (Huque et al., 2018). All assumptions were checked and found to be satisfactory.

To address our second research question, we employed linear mixed models (Bates et al., 2015) with adolescents’ daily mindset as the dependent variable. This method is particularly suited for diary data (Nezlek, 2012), as it appropriately accounts for the nested structure of day-level measurements within participants, thereby addressing issues of dependency and within-subject variability (Snijders & Bosker, 1999). For each adolescent–parent dyad, data on two levels were available, at the within-participant level (level 1; daily process- and result-oriented parental feedback) and at the between-person level (level 2; demographic variables, such as gender and age of the adolescent and of the parent). The within-participants level is nested within the between-person level data and was modeled as a random effect using maximum likelihood estimation. Between person-level predictor variables were centered around the grand mean (gender and age of the adolescent and gender of the parent), and within-level predictor variables were centered around the respective person mean (feedback provided by parents) (see, e.g., Sonnentag, 2003). To establish whether there was sufficient unexplained variance at the within-person level, we calculated intraclass correlations for percentages of between and within-person variance. For this purpose, we first estimated the intercept-only model for adolescents’ intelligence mindset (the null model that contains no explanatory variables). Next, we entered level-1 fixed effects. These included process-oriented and result-oriented feedback provided by parents. Our third step was to include level-2 fixed effects; the demographic variables (gender and age of the adolescent and gender of the parent). In the last step, we added random slopes of process and result-oriented feedback provided by the parents. This will account for the possibility that individuals have different rates of change in the dependent variable (Hox, 2000). The goodness of fit of the different models was evaluated using several fit indices and diagnostic checks. Given the limited sample size of the dataset in this study, Bayesian Information Criterion (BIC) sensitivity to sample size could result in an undue penalization of more complex models, potentially leading to underfitting. Therefore, we opted not to report BIC and instead focused on Akaike Information Criterion (AIC) and the deviance difference (ΔD*)* based on a chi-square distribution.

## Results

### Descriptive statistics

Table 3 presents the mean, standard deviations, and correlations of the different measures taken in the cross-sectional part of this diary study (*N* = 129). We identified a significant positive correlation between parents’ mindsets and their adolescents’ mindsets, indicating that parents with a more growth mindset tended to have adolescents who also exhibited a more growth mindset (*r* =.378, *p* <.001). Additionally, a positive correlation was observed between parents’ failure beliefs and their mindsets (*r* =.388, *p* <.001), suggesting that parents who viewed failure as enhancing more often had a growth mindset.

**Table 3:** Mean, standard deviations, and correlation of the different measurements of parent–adolescent dyads (*N* = 129)

	Mean	SD	1	2	3	4
1. Mindset (A)^a^	4.62	.73				
2. Mindset (P)	4.39	.84	.378^b^			
3. Failure beliefs (P)	2.85	.85	−.142	.388^b^		
4. Appraisal of achievement–Increasing marks (P)	3.36	.58	−.030	−.028	.044	
Covariates						
5. Age (A)	14.47	2.00	−.299^b^	−.108	−.049	.049
6. Age (P)	47.60	4.58	−.090	−.039	.068	.117
	Male:female	Missing				
7. Gender (A)	50:68	11	−.147	−.078	.036	.016
8. Gender (P)	30:94	5	.052	−.051	.137	.078

*(A)* measure reported by the adolescent, *(P)* measure reported by the parent

^a^Higher scores (max 6) indicate a more growth mindset, while lower scores (min 1) indicate a more fixed mindset

^b^Correlation is significant at the 0.05 level

In the regression analysis, we found a significant direct effect of the mindset of parents on the mindset of adolescents (*β* =.351, 95% CI [0.152–0.491]), suggesting that parents with a more growth mindset had adolescents with a more growth mindset as well (see Table 4). We found no direct significant effect of the failure beliefs of parents on adolescents’ mindset (*β* = − 0.017, 95% CI [− 0.183–0.152]). The covariate age of the adolescents significantly predicted adolescents’ mindset (*β* = − 0.262, 95% CI [− 0.165 to − 0.342]), indicating that older adolescents had a more fixed mindset than younger adolescents. Other covariates (age of the parent, gender of the adolescent, and gender of the parent) did not reveal any significant effects.

**Table 4:** Regression analysis of parents’ intelligence mindset and parents’ failure beliefs on adolescents’ mindset (*N* = 129 parent–adolescent dyads)

Variables	*β*	SE	95% CI		*p*
			*LL*	*UL*	
Intelligence mindset (P)	0.351	0.103	0.152	0.491	<.001^a^
Failure beliefs (P)	− 0.017	0.085	− 0.183	0.152	.856
Appraisal of increasing marks (P)	− 0.031	0.121	− 0.274	0.190	.720
Gender adolescent	− 0.109	0.134	− 0.428	0.095	.210
Age adolescent	− 0.262	0.033	− 0.165	− 0.342	.003^a^
Gender parent	0.065	0.163	− 0.186	0.408	.459

(P) is measurement taken from the parent; gender is coded as 0 = male and 1 = female. *F*-statistics; *F*(6, 103) = 5.456, *p* <.001

*CI* confidence interval, *LL* lower limit, *UL* upper limit

^a^Regression is significant at the.05 level

### The mediating role of appraisal of increasing marks

**Fig. 1:**
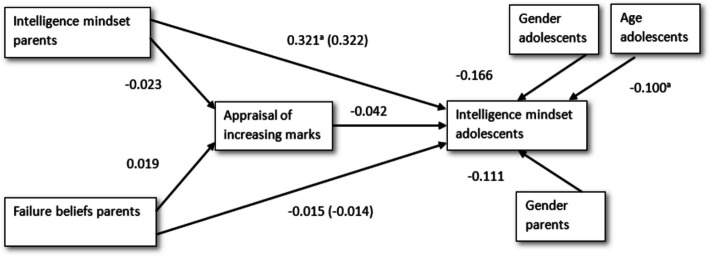
Standardized regression coefficients for the association between parental beliefs (intelligence mindset and failure beliefs) and adolescents’ intelligence mindset, as mediated by appraisal of increasing marks, controlling for gender of the adolescent and gender of the parent. ^a^Significant at the 0.05 level

Results for the mediation analysis are depicted in Fig. 1. Within the mediation analysis, the regression of parental intelligence mindset on the mediator, appraisal of increasing marks, was not significant (*β* = − 0.023, 95% CI [− 0.178–0.144] (a_1_)). Further, the regression of parental failure beliefs on appraisal of increasing marks was not significant (*β* = − 0.019, 95% CI [− 0.166–0.191] (a_2_)). Appraisal of increasing marks, when controlling for gender and age of the adolescents and gender of the parents, did not significantly predict adolescents’ mindset (*β* = − 0.042, 95% CI [− 0.270–0.204] (b)).

Unstandardized indirect effects were computed for each 5000 bootstrapped samples. The indirect effect for parents’ mindset was *b* = 0.001 (a_1_b). We tested the significance of this effect using bootstrapping procedures. The 95% confidence interval computed by determining the indirect effects at the 2.5th and 97.5th% and ranged from − 0.013 to 0.037. The indirect effect was statistically non-significant (*p* =.781), showing that appraisal of increasing marks did not mediate the relationship between parents’ mindset and adolescents’ mindset.

Additionally, the indirect effect for parents’ failure beliefs was *b* = 0.001 (a_2_b). The 95% confidence interval ranged from − 0.034 to 0.018. This indirect effect was also statistically non-significant (*p* = 0.755), indicating that appraisal of increasing marks did not mediate the relation between parents’ failure beliefs and adolescents’ mindset.

To assure the validity of these mediation effects, Robus mediation (ROBMED) analysis proposed by Alfons et al. (2022a) was applied. ROBMED uses the first linear regression in testing mediation. Second, it utilizes the state-of-the-art method of bootstrapping for testing the indirect effect in mediation models. After treating the two possible outliers, following recommendations by Aguinis et al. (2013), the results of the ROBMED confirmed our findings.

### Parental feedback and daily fluctuation in adolescents’ mindset

Table 5 presents the results of the stepwise mixed model analysis with which we investigated whether daily process or result-oriented feedback of parents was related to day-to-day fluctuations in adolescents’ mindset. To establish whether there was sufficient unexplained variance at the within-person level, that is, whether there were daily fluctuations within persons, we first calculated the intraclass correlations. For this, we estimated the intercept-only model for adolescents’ mindset without any explanatory variables. The intraclass correlation was.715 for adolescents’ mindset across the ten days, demonstrating that 71.5% of the variance was explained by mindset differences between adolescents, and 29.5% of the variance was explained by daily fluctuations in mindset within adolescents.

**Table 5:** Linear mixed model with intercept only (model 1), fixed level-1 (model 2), and fixed level-2 predictors (model 3) (*N* = 122)

	Model 1 (intercept-only)			Model 2			Model 3		
	*b*	*SE*	*p*	*b*	*SE*	*p*	*b*	*SE*	*p*
Intercept	2.892	0.104	<.001^a^	2.892	0.104	<.001^a^	2.892	0.099	<.001^a^
Within adolescents									
Result-oriented feedback^b,c^				− 0.121	0.493	.014^a^	− 0.121	0.049	.014^a^
Process-oriented feedback^b,c^				0.033	0.051	0.514	0.034	0.051	.514
Between variables									
Age of the adolescent^d^							0.107	0.048	.028^a^
Gender of the adolescent^d^							0.285	0.201	.159
Gender of the parent^d^							− 0.481	0.233	.042^a^
AIC	3030.0			3027.8			3023.3		
Log likelihood	− 1512.0			− 1508.9			− 1503.7		
Deviance difference				7			17		
Degrees of freedom				*df* = 2			*df* = 3		
				(*p* =.044^a^)			(*p* =.015^a^)		
ICC	0.715								

^a^Regression is significant at the.05 level

^b^Data is centered around the person mean

^c^Measured in parents

^d^Data is centered around the grand mean

We first added parental result-oriented feedback and process-oriented feedback as level-1 fixed effects (model 2). To evaluate the model fit, we used AIC and deviance difference. The model including level-1 fixed effects showed a lower AIC (AIC = 3027.8) compared to the intercept-only model (AIC = 3030.0), indicating an improved balance between goodness-of-fit and model complexity. The deviance difference test indicated a significant improvement in model fit (*ΔD* = 7, *p* =.044), based on a chi-square distribution with 2° of freedom. These results confirm that model 2 provides a significantly better fit than the intercept-only model. Model 2 shows an effect of result-oriented feedback (*b* = − 0.121, *t*(122) = − 2.455, *p* =.014) within participants, suggesting that on days on which parents provided their adolescents with more feedback based on their achievements, their adolescents had a more fixed mindset than on days that parents provided less feedback based on achievements. We did not find this effect for process-oriented feedback.

Second, we added gender and age of the adolescent and gender of the parent as level-2 fixed effects (model 3). This model demonstrates a significant improvement in fit compared to model 2, as indicated by a lower AIC value (AIC in model 3 = 3023.3 versus 3027 in model 2), indicating better model parsimony. Furthermore, the deviance difference test confirmed this improvement, with a deviance difference of *ΔD* = 17 (*p* =.015), which was statistically significant based on a chi-square distribution with 3° of freedom. These results significantly enhanced the model’s explanatory power. Model 3 shows an effect of age of the adolescent, suggesting that older adolescents had a more growth mindset than younger adolescents (*b* = 0.048, *t*(122) = 2.231, *p* =.028). We also found an effect for gender of the parent, suggesting that adolescents who participated together with their mothers in this investigation had a more fixed mindset than adolescents who participated with their fathers (*b* = − 0.121, *t*(122) = − 2.455, *p* =.014).

In our last step, we included random slopes of process-oriented and result-oriented feedback provided by the parents. The model showed a higher AIC (*AIC* = 3031.6) compared to model 3 (*AIC* = 3023.3), suggesting that adding random slopes did not significantly improve our model. Thus, the associations between day-to-day parental feedback and daily fluctuations of adolescents’ mindset did not differ between individual adolescents. This poor fit was confirmed by the deviance difference test, which shows that the deviance difference is *ΔD* = 1,7 (*p* =.88) compared to model 3, based on a chi-square distribution with 5° of freedom. Consequently, we chose not to report the results from this model, as they could lead to misleading conclusions regarding the relationship between parental feedback and adolescents’ mindset. Our final model, model 3, explained 50.1% of the total variance in adolescents’ mindset. Regression coefficients are presented in Table 5.

## Discussion

In this study, we first explored in a cross-sectional study how parental beliefs, such as parents’ mindset and parents’ failure beliefs, relate to adolescents’ mindset and whether this effect was mediated by parents’ appraisal of increasing marks. Second, we explored how day-to-day parental feedback, focusing on either the process or the results of their adolescents’ schoolwork, was related to daily fluctuations in adolescents’ mindset. In the cross-sectional study, our findings indicated that parental mindset of intelligence, but not parents’ failure beliefs, was positively related to adolescents’ mindset. Parents’ appraisal of increasing marks did not mediate the relationship between parents’ intelligence mindset and failure beliefs and adolescents’ intelligence mindset. In the diary study, we found that, on days with higher levels of parents’ feedback focused on their adolescents’ results, adolescents reported a more fixed mindset. Unexpectedly, we did not find that day-to-day parental feedback focused on the process of learning was related to their adolescents having a more growth mindset. We discuss each of these findings and their implications in more detail below.

### Parental beliefs and adolescents’ mindset

Even though previous research reported conflicting findings regarding the relationship between parents’ intelligence mindset and adolescents’ intelligence mindset, we found a positive relationship. This implies that parents with a more fixed mindset have adolescents with a more fixed mindset, while parents with a more growth mindset have adolescents with a more growth mindset. Previous investigations by Gunderson et al. (2018) and Haimovitz and Dweck (2016) did not find this relationship. Haimovitz and Dweck (2016) suggested that parents’ mindset itself may not be very visible to their children, and therefore, children do not necessarily develop the same mindset as their parents. However, other investigations found a positive relationship between parents’ mindset and adolescents’ mindset (Tian, 2019) or suggested that parents’ growth mindset is related to growth mindset-related characteristics in adolescents, including high persistence and high motivation (Jose & Bellamy, 2012; Pomerantz & Dong, 2006). Our findings are also in line with the recently developed process model of mindsets (PMM), according to which parents may have a strong impact on their adolescents’ mindsets due to their authority, and adolescents have a tendency to adapt their beliefs to be more similar to those they want to maintain a bond with. According to the PMM, adolescents develop their mindset situated in the social context. Evidence for this comes from studies in other social contexts, such as school. For example, a study by Muenks et al. (2020) showed that adolescents in a school environment, who were characterized by a growth mindset, experienced a greater sense of belonging and felt more comfortable making mistakes. Altogether, our findings confirm the importance of the social environment for adolescents to develop a more growth mindset, and that this social influence can also come from parents.

Contrary to our hypothesis, we did not find a relationship between parents’ failure beliefs and their adolescents’ mindset. Parents who see failure as debilitating do not necessarily have adolescents with a more fixed mindset, while parents who see failure as a learning opportunity do not necessarily have adolescents with a more growth mindset. Previous studies did find this relationship (Haimovitz & Dweck, 2016; Peterson et al., 2024; Tao et al., 2022); however, none of these studies controlled for parents’ mindset of intelligence. Our study controlled for parents’ intelligence mindset, which may have contributed to the lack of a statistically significant relationship between parents’ failure beliefs and adolescents’ intelligence mindset. Another potential explanation for our non-significant findings could be the use of quantitative data in our study. It is possible that qualitative data, as collected and analyzed in an experiment carried out by Peterson et al. (2024), may be more appropriate for investigating failure beliefs, as it allows for a deeper exploration of individual perceptions and experiences. Carrying out experiments with a more qualitative research design to gather more in-depth data may be an important next step in future research.

### The mediating role of appraisal of increasing marks

Contrary to our hypothesis, our findings did not show a mediation effect of appraisal of increasing marks on the relationship between parental beliefs and adolescents’ mindset. Parents with a more growth mindset or parents who see failure as a learning process did not appraise increasing marks more, and this was not related to their adolescents’ mindset. Kraker-Pauw et al. (2017) in their study found a relationship between teachers’ growth mindset and their appraisal of increasing marks. However, the fact that we were unable to find an effect of appraisal of increasing marks could be because of the different roles teachers and parents have. As a teacher, it is important to have a growth mindset and to see failure as a learning process by focusing on individual progress such as increasing marks, in order to make adolescents persist in their school tasks and to keep them motivated. Parents might have different interests and stakes than teachers. At the end of the school year, it is not the process of learning that is the most important factor for determining whether an adolescent is allowed to continue the same academic track, but school results (marks) are. Therefore, for parents who want their adolescents to succeed and to continue at the same educational level, not the appraisal of increasing marks, but sufficient final results, may be equally or even more important.

Another factor that may have influenced our findings is the methodology used in this study. We asked parents to evaluate the third mark from 12 sets of three sequential marks obtained by a fictional adolescent. It is possible that teachers may be more accustomed than parents to assessing and interpreting a series of obtained marks, and it is possible that parents may have automatically projected the sets with marks on their own adolescent. This difference in familiarity may have contributed to the absence of a relationship between increasing marks and a growth mindset in our study, whereas similar studies involving teachers did observe such a relationship. Utilizing an alternative measure to examine the relationship between parents’ appraisal of increasing marks and their adolescents’ mindset may provide valuable insights and warrant further investigation.

### Parental feedback and daily fluctuations in adolescents’ mindset

In the diary study, we tested whether day-to-day parental feedback was related to daily fluctuations in the mindset of their adolescents. According to the PMM (de Ruiter & Thomaes, 2023), mindsets are thought to have both trait and state components. The trait component refers to an individuals’ general belief about the nature of intelligence, whether intelligence is fixed or malleable. The state component, on the other hand, is more dynamic and may fluctuate based on situational factors. As far as we know, there are no previous studies that looked at daily fluctuations in mindset. A few existing studies have primarily focused on fluctuations in mindset over a longer period of time, such as an academic semester or multiple school years (Costa & Faria, 2023; Limeri et al., 2020; Shively & Ryan, 2013). One study indicated that students’ implicit beliefs about intelligence remained stable throughout secondary school (Costa & Faria, 2023). In contrast, two other investigations observed a trend toward a reduced growth mindset, with students increasingly perceiving intelligence as a stable trait, demonstrating a shift toward a stronger fixed mindset and a weaker growth mindset over time (Limeri et al., 2020; Shively & Ryan, 2013). However, in most studies, mindset is approached as stable trait, and the situational component is largely overlooked. Some studies have demonstrated fluctuations in motivational beliefs, providing evidence for their dynamic nature. For instance, Neubauer et al. (2022) identified day-to-day state-like variations in achievement goals. Similarly, a study by Waninge et al. (2014) revealed that motivation exhibits temporal changes at the individual level. Our study showed that mindset is indeed fairly stable within persons. The situational component, i.e., the daily variations within persons, seemed small by comparison. Although the trait component was strong, the mindset still seemed to have some variability across days. This duality highlights that, while individuals may generally lean toward a fixed or growth mindset (trait), their mindset can temporarily shift depending on daily experiences or specific challenges.

When looking at these findings from the perspective of ecological systems theory (Bronfenbrenner, 1979), individuals develop within a series of nested environmental systems that interact dynamically over time. This framework emphasizes that beliefs, such as mindsets, are not fixed in isolation but are continually shaped by contextual influences. Building on this ecological view, recent theoretical and empirical work has emphasized the importance of the social context—particularly the roles of parents, teachers, and peers—in shaping mindset development (de Ruiter & Thomaes, 2023; King, 2020; Lou & Li, 2023; Yu et al., 2022).

This process model of mindsets (PMM), developed by de Ruiter and Thomaes (2023), extends Dweck’s original theory and conceptualizes mindset as a dynamic, (social) context-sensitive state rather than a fixed individual trait. The PMM proposes that mindsets fluctuate along a fixed-to-growth continuum, influenced by situational factors such as feedback, task difficulty, and prior experiences. In line with Bronfenbrenner’s theory, the PMM highlights that these fluctuations are embedded within—and shaped by—the broader ecological systems surrounding the individual. Together, both these models support a view of mindset as an adaptable belief system, continuously influenced by the social environment.

Developing this further, de Ruiter et al. (2020) introduced a more practical framework for analyzing how person-specific mindset-related language fluctuates during between teacher and student interactions in a classroom. Their findings showed that teachers and students engaged predominantly in language associated with a fixed mindset throughout the interactions. Additionally, teachers and students revealed a strong sequential association suggesting that their mindset-related verbalizations were closely aligned over time. However, neither the teacher nor the student consistently leads in initiating fixed- or growth-oriented language. This dynamic shift in leadership over mindset-related language between teachers and students may also play a crucial role in relation to influential others with whom adolescents interact in their environment, such as their parents.

Parents of adolescents may shape how adolescents respond to learning. These interactions may take different forms, such as offering feedback on achievements or on the process. For example, acknowledging a person for their hard work may promote a more growth mindset, whereas providing feedback based on results may contribute to a more fixed mindset, in particular when this feedback comes from an individual with authority, such as a teacher or a parent. Our findings regarding the role of daily parental feedback in situational mindsets add to our understanding of mindset development in daily situations. We found, in line with our hypothesis, a negative relationship between result-oriented feedback provided by parents during day-to-day interactions and adolescents’ mindset. Parents providing feedback based on their adolescents’ actual school results during day-to-day interactions was associated with adolescents exhibiting a more fixed mindset on that day. Contrary to our hypothesis, we found no significant relationship between process-oriented feedback and fluctuations in adolescents’ mindset. Feedback that emphasizes results rather than the learning process is recognized as less effective in fostering adaptive mindsets (Hattie & Timperley, 2007; Shute, 2008). However, in our study, the extent of process-oriented feedback did not affect daily fluctuations in adolescents’ mindset. A similar finding was reported by Pomerantz and Kempner (2013), who observed that mothers’ daily use of person praise was associated with a fixed mindset in children, whereas process praise was not significantly related to a growth mindset. Our findings may be of special interest to parents who provide their adolescents with feedback based on achievements, as they may unintentionally raise their adolescents to develop a more fixed mindset, which consequently might affect their adolescents to have lower intrinsic motivation and academic achievements, and therefore less academic resilience and success. Additionally, the adjustment of mindset does not have to be a long-term process, since our study shows that this adaptation already takes place during day-to-day interactions between parents and their adolescents. Understanding the dynamic interplay between a stable trait and received parental feedback may be critical in interpreting how mindsets may shift in different social contexts.

Regarding the type of feedback parents provide their adolescents with, it is possible that process-oriented feedback may not directly relate to a growth mindset, as this type of feedback may be less salient than result-oriented feedback. It is also possible that process-oriented feedback needs to be more frequent, specific, and linked to effort and strategies in order for adolescents to develop a more growth mindset. Research by Dweck (1999) shows that, when feedback emphasizes effort and strategies (rather than innate ability or just outcomes), it helps students develop a growth mindset—the belief that intelligence and abilities can be developed through effort and learning. However, for this to work, Dweck first states that feedback must be frequent to reinforce the value of effort over time. Second, it must be specific, pointing out exactly what the student did well in terms of strategy or persistence (e.g., “You broke the problem into smaller parts and that helped you understand it better.”), and lastly, it must be linked to controllable factors, like effort and strategy, not fixed traits (Burnett, 2003). Adolescents, in particular, benefit from explicit connections between effort, strategy, and improvement, as they are still developing metacognitive skills and a sense of agency over their learning (Zimmerman, 2002).

Additionally, adolescents may be more sensitive to person praise than younger children or adults, as during the phase of adolescence, they develop their own values and beliefs (Dent & Koenka, 2016; Wesarg-Menzel et al., 2023). However, since result-oriented feedback provided by parents relates to a fixed mindset in their adolescents, an important next step will be to make parents aware of the feedback they provide to their adolescents, particularly when they focus more on results, and the important role this has in the development of a fixed mindset in their adolescents. Moreover, understanding how result-oriented and process-oriented feedback relate to the stability and contextual sensitivity of mindset may offer a valuable foundation for developing analytical approaches to studying parent–adolescent interactions.

## Strengths and limitations

Our study has several strengths. First, this study is the first that assess a situational component, as it includes day-to-day fluctuations in adolescents’ mindset and their relationship with parental feedback. As such, our study provides important contributions to the PMM model of mindset. Second, we were able to collect data from one parent and their adolescent, which is particularly challenging for this age group. This dyadic data made it possible to measure (self-reported) interaction between parents and their adolescents, and this contributes to a better understanding of the interaction between parents and their adolescents compared to using only measurements from either the adolescent or the parent. Additionally, we were able to collect data from a significant number of fathers. Their inclusion improves the representativeness of the sample and enhances the generalizability of our findings to fathers as well as to mothers.

However, a number of limitations should be mentioned as well with regard to the current study. First, a limitation of this study is our cross-sectional design. While cross-sectional data can provide initial insights into associations between variables, it does not allow for conclusions about the temporal ordering of effects. In particular, mediation analyses are best conducted using longitudinal data, which can account for the stability of the dependent variable over time. Second, due to the observational nature of the data, in our study, we were unable to provide causal inferences from this data. It would be useful for future studies to use a more experimental research design to get a better understanding of the causal mechanisms behind parents’ influence on fluctuations in adolescents’ mindset. Third, while it is a strength to include data from both parents and their adolescents, the recruitment of both of them presented additional practical hurdles relative to studying individuals. This contributed to the relatively small sample size of our study. Fourth, to get a more in-depth view of adolescents’ mindset and parental feedback, future studies should go beyond self-report and combine daily questionnaires with observations or interviews with both parents and their adolescents in order to understand how parental daily feedback may influence the possible shifts in adolescents’ mindset. Last, the adolescents and parents in this study predominantly came from an affluent region in the Netherlands. As such, the generalizability of our findings to other (sub)populations may be limited. Prior research in the Netherlands has indicated that children from socioeconomically advantaged backgrounds typically receive substantially more parental support than those from disadvantaged backgrounds (Bol, 2020). This suggests that parental involvement and related outcomes may differ across social groups. Consequently, future research should also include families from less affluent regions within the Netherlands and from diverse international settings to gain a more comprehensive understanding.

## Conclusion

In sum, this study examined the role of parental intelligence mindset and failure beliefs, their appraisal of increasing marks, and the feedback they provided on a daily basis, on their adolescents’ mindset in a combined cross-sectional and daily diary study with parents and their adolescents. Our findings revealed a positive relationship between parents’ intelligence mindsets and their adolescents’ intelligence mindsets. Adolescents were more likely to exhibit a growth mindset when their parents also demonstrated a growth-oriented mindset. Importantly, we found that parents’ day-to-day result-oriented feedback was negatively associated with daily fluctuations in adolescents’ fixed intelligence mindset, indicating that a focus on actual school marks rather than the learning process may hinder the development of a growth mindset in adolescents. These findings have useful implications, such as providing insights into the importance of parental feedback during day-to-day interactions. Moreover, this parental feedback may be crucial to understand how a relatively stable trait such as intelligence of mindset may shift across different social contexts. Parental intelligence beliefs and the feedback they provide may play a crucial role in fostering adolescent motivation and promoting their persistence and may potentially shape the development and adjustment of adolescents’ mindsets.

## Data Availability

The datasets used and/or analyzed during the current study are available from the corresponding author upon reasonable request.
